# Supplementary material for: Social Determinants of Stroke Hospitalization and Mortality in United States’ Counties
Source: J Clin Med. 2022 Jul 15;11(14):4101. doi: 10.3390/jcm11144101 (PMC9320068; doi:10.3390/jcm11144101)

<u>Supplementary Materials</u>

Social Determinants of Stroke Hospitalization and Mortality in United States' Counties

Table of Content

1. **Table S1.** Studies showing associations of socioeconomic variables with stroke incidence and outcomes in the United States.
2. **Table S2.** Name, definitions, and data sources of variables used in the study.
3. **Table S3.** Modeling Log standardized hospitalization ratio weighted by expected counts for each county.
4. **Table S4.** Modeling Log standardized mortality ratio weighted by expected counts for each county.
5. **Figure S1.** Correlation matrix and heatmap of predictor variables used in the study.
6. **Figure S2.** Map of US counties and county equivalents showing A. Prevalence of heart disease among Medicare beneficiaries, B. Blood pressure medication non-adherence, C. Cost of care among Medicare beneficiaries with heart disease, D. Age-adjusted obesity, E. Presence of hospital with neurological services, F. Presence of hospital.

Table S1. Studies showing association of socioeconomic factors with stroke incidence and outcomes in the United States.

**Author**	**Objective/Purpose** (to evaluate)	**Methods/Design**	**Study Population**	**Variables studied**	**Results/Findings**
(Avendano et al. 2006) [3]	SES on stroke incidence in the elderly	Aged ≥65 years, followed for 12 years. Proportional hazard models were applied.	≥65 years from the New Haven cohort of EPESE	Education, income, Hypertension, Smoking, Diabetes, Body mass index, Physical activity, Alcohol consumption, Physical/Cognitive Functioning, Psychosocial Risk Factors	N= 2812. At ages 65 to 74, LSES: education (HR: 2.07; 95% CI: 1.04-4.13), income (HR: 2.08; 95% CI: 1.01-4.27). Cross over association in >75 year.
(Avendano and Glymour 2008) [4]	Independent effect of wealth, income, and education on stroke incidence in middle and old age	Stroke-free participants were followed 8.5 years. Cox proportional hazard models to predict time to stroke.	Health and Retirement Study (HRS)- national sample of US adults aged ≥50 and their spouses	Household wealth, household income, and education, smoking status, body mass index (BMI), vigorous physical activity, hypertension, diabetes, and heart disease	N= 19,565. Wealth and income were independent risk factors for stroke at ages 50-64 but not in≥65 years.
(Bettger et al. 2014) [5]	Education, work, and income with disability 3-months following acute ischemic stroke.	Retrospective analyses of a prospective cohort. Multivariable logistic regression was	≥18 years hospitalized at a GWTG-Stroke participating hospital with a primary diagnosis of transient ischemic attack or ischemic stroke based	education, income and employment	In survivors, after adjusting; unemployed, not-working, retired, less educated, or inadequate income prior to stroke had a significantly higher

		applied.	on ICD-9		odds of post-stroke disability.
(Hanchate et al. 2013) [6]	Inpatient mortality and length of stay by race/ethnicity and SES in eight US states	Population was stratified by race/ethnicity (non-Hispanic whites, non-Hispanic blacks, and Hispanics) and SES, measured by median income of patient zip code.	Acute ischemic stroke admissions for adults ≥18 years; state-level inpatient discharge databases from all nonfederal acute care hospitals in Arizona, California, Florida, Maine, New Jersey, New York, Pennsylvania, and Texas.	race/ethnicity, socioeconomic status, measured by median income of patient zip code, patient risk factors	Adjusted inpatient mortality was similar by race/ethnicity, but was significantly higher for low-income area (OR: 1.08; 95%CI: 1.02-1.15). Risk-adjusted longer length of stay higher in minority and low-income area.
(Howard et al. 2016) [7]	Neighborhood socioeconomic characteristics and incident stroke in a national cohort of black and white participants.	National population-based cohort was done.	≥45 years, enrolled in 2003–2007 in Reasons for Geographic and Racial Differences in Stroke (REGARDS)	Neighborhood SES (nSES), Educational level, Annual household income, Framingham stroke risk factors	After adjustment; stroke incidence increased with each decreasing nSES quartile. HR; Q3=1.28 (95%CI: 1.05–1.56), Q2=1.38 (95%CI: 1.13–1.68), Q1=1.56 (95%CI: 1.26–1.92) (p=0.0001). Marginally significant for individual SES; attenuated the association between nSES and stroke in both

					black and white participants, greater in blacks
(Pedigo, Aldrich, and Odoi 2011) [8]	Spatial patterns of stroke and MI mortality risks in the East Tennessee to identify neighborhoods with the highest risks.	Logistic models were used to investigate neighborhood level socioeconomic and demographic predictors of the identified spatial clusters.	11 counties of the East Tennessee Appalachian Region that have some of the highest risks of stroke and/or MI in the state-Census tracts (CTs)	black race, gender, age 65 years and older, household income, education less than high school, population below poverty, median housing value, geography (urban versus rural), employment, single parent families, marital status, and housing ownership,	Neighborhoods belonging to high risk clusters of stroke and MI mortality tended to have high proportions of the population with low education attainment.
(Brown et al. 2011) [9]	Neighborhood SES and incident ischemic stroke and potential mediators of these associations.	The Cardiovascular Health Study, a multicenter, population-based, longitudinal. Race-stratified multilevel Cox proportional hazard	Whites and blacks enrolled in the Cardiovascular Health Study- ≥65 years from 4 US counties.	Neighborhood SES, age, sex, race, median household income, and education, smoking history, physical activity, alcohol use, and diet, cardiovascular disease, atrial fibrillation, hypertension, diabetes, and total cholesterol to high density lipoprotein	Adjusted: in whites, stroke hazard significantly higher in neighborhoods in lowest SES quartile (HR, 1.32; 95% CI, 1.01–1.72) with greater attenuation after adjustment for biological risk factors (HR, 1.16; 0.88–1.52) than for behavioral risk

				cholesterol ratio	factors (HR, 1.30; 0.99–1.70). No significant associations in blacks
(Schieb et al. 2013) [10]	Clustering of stroke hospitalization rates, patterns of the clustering over time, and associations with community-level characteristics	Medicare hospital claims data from 1995-1996 to 2005-2006 with a principal discharge diagnosis of stroke to calculate county-level stroke hospitalization rates.	Medicare beneficiaries aged ≥65	age, sex, and race or ethnicity, Median household income, poverty, without a high school diploma, Health care, Urban/rural, Migration	Persistently low-stroke hospitalization rates counties had the most favorable socioeconomic and healthcare profiles, followed by counties that transitioned out of or into high-stroke hospitalization rates clusters. Persistently high-stroke hospitalization rates counties experienced the least favorable socioeconomic and healthcare profiles.

N: sample size; SES: Socioeconomic status; LSES: Low Socioeconomic status; HR: Hazard Ratio; RR: Relative Risk; OR: Odds Ratio; CI: Confidence Interval, Q: quartile.

Table S2. Name, definitions, and data sources of variables used in the study.

Variables	Definitions	Data Sources
Stroke Hospitalization Rate (SHR)	All Stroke Hospitalization Rate per 1,000 Medicare Beneficiaries, 65+, All Races/Ethnicities, Both Genders, 2015-2017	Centers for Medicare and Medicaid Services Medicare Provider Analysis and Review (MEDPAR) file, Part A.<br>http://www.cms.gov/Research-Statistics-Data-and-Systems/Statistics-Trends-and-Reports/MedicareFeeforSvcPartsAB/MEDPAR.html
Stroke Death Rate (SDR)	All Stroke Death Rate per 100,000, All Ages, All Races/Ethnicities, Both Genders, 2016-2018<br><br>All Stroke: I60-I69; underlying cause of death.<br>Ischemic Stroke: I63, I65-I66; underlying cause of death.<br>Hemorrhagic Stroke: I60-I62; underlying cause of death.	Deaths National Vital Statistics System. Bridged-Race Postcensal Population Estimates from National Center for Health Statistics.<br>http://www.cdc.gov/nchs/deaths.htm<br>http://www.cdc.gov/nchs/nvss/bridged_race.htm
Park access	Percentage of Population Living Within Half a Mile of a Park, 2015	CDC Environmental Public Health Tracking Network<br>Original data sources: NAVTEQ and U.S. Census Bureau<br>http://ephtracking.cdc.gov/showAccessToParksAndSchools.action
Hospital present	Hospitals, 2017	Health Resources and Services Administration: Area Health Resources File<br>https://datawarehouse.hrsa.gov/topics/ahrf.aspx
Hospitals with Neurological services	Hospitals with Neurological Services, 2017	Health Resources and Services Administration: Area Health Resources File<br>https://datawarehouse.hrsa.gov/topics/ahrf.aspx
Age-adjusted obesity	Obesity, Age-Adjusted Percentage, 20+. 2016	CDC Division of Diabetes Translation: CDC Behavioral Risk Factor Surveillance System, U.S. Census Bureau Population Estimates

		http://www.cdc.gov/diabetes/data/county.html
Without college	Percentage without 4+ Years College, Ages 25+, 2014-2018 (5-year)	U.S. Census Bureau (ACS): American Community Survey, (5-year estimate)<br>http://factfinder2.census.gov/
Age-adjusted diabetes	Diagnosed Diabetes, Age-Adjusted Percentage, 20+, 2016	CDC Division of Diabetes Translation: CDC Behavioral Risk Factor Surveillance System, U.S. Census Bureau Population Estimates<br>http://www.cdc.gov/diabetes/data/county.html
Age-adjusted physical Inactivity	Leisure-Time Physical Inactivity, Age Adjusted Percentage, 20+, 2016	CDC Division of Diabetes Translation: CDC Behavioral Risk Factor Surveillance System, U.S. Census Bureau Population Estimates<br>http://www.cdc.gov/diabetes/data/county.html
Income inequality	Income Inequality (Gini Index), 2014-2018 (5-year)	U.S. Census Bureau (ACS): American Community Survey, (5-year estimate)<br>http://factfinder2.census.gov/
Unemployment	Unemployment Rate, Ages 16+, 2018	U.S. Bureau of Labor Statistics: Local Area Unemployment Statistics<br>http://www.bls.gov/
Female head of household	Families with Female Head of Household (%), 2014-2018 (5-year)	U.S. Census Bureau (ACS): American Community Survey, (5-year estimate)<br>http://factfinder2.census.gov/
Food assistance	Percentage Food Stamp/Supplemental Nutrition Assistance Program Recipients, 2016	Health Resources and Services Administration: Area Health Resources File<br>https://datawarehouse.hrsa.gov/topics/ahrf.aspx
Household income	Median Household Income (in thousands of $), 2018	U.S. Census Bureau (SAIPE): Small Area Income and Policy Estimates<br>https://www.census.gov/programs-surveys/saipe.html
Poverty	Percentage Living in Poverty, All Ages, 2018	U.S. Census Bureau (SAIPE): Small Area Income and Policy Estimates<br>https://www.census.gov/programs-surveys/saipe.html
Cost of care	Cost of Care per Capita for Medicare	Centers for Medicare and Medicaid Services' (CMS) Chronic

	Beneficiaries Diagnosed with Heart Disease, Outpatient, 2017	Conditions Data Warehouse (https://www.cdc.gov/dhdsp/maps/atlas/maps-of-cost-data.html
Heart disease	Prevalence of Diagnosed Heart Disease Among Medicare Beneficiaries, 2017	Centers for Medicare & Medicaid Services: Chronic Conditions Data Warehouse https://www.ccwdata.org/
Blood pressure medication nonadherence	Blood Pressure Medication Nonadherence Percentage, All Races/Ethnicities, Medicare Part D Beneficiaries Aged 65+, 2017<br><br>Medication Nonadherence, defined as a proportion of days a beneficiary was covered with medication of <80%, was assessed using prescription drug claims data among Medicare Advantage or Medicare fee-for-service beneficiaries aged ≥65 years with Medicare Part D coverage.	Medicare Part D Claims Data: Centers for Medicare & Medicaid Services, CDC Vital Signs Report https://www.cdc.gov/vitalsigns/blood-pressure

Table S3. Modeling Log standardized hospitalization ratio weighted by expected counts for each county.

**Variable**	**Estimate (95% CI)**	***p-value***
Prevalence of heart disease among Medicare patients	0.06 (0.04 - 0.07)	<0.001
Food assistance	0.005 (-0.01 - 0.02)	0.52
Age-adjusted obesity	0.005 (0 - 0.02)	0.331
Female head of household	0.03 (0.01 - 0.05)	<0.001
Blood pressure medication nonadherence	0.03 (0 - 0.05)	0.014
Age-adjusted physical inactivity	0.01 (0 - 0.02)	0.099
Cost of care among Medicare patients with heart disease	-0.08 (-0.09 - -0.07)	<0.001
Age-adjusted diabetes	0.01 (0 - 0.02)	0.095
No college degree	0 .001 (-0.01 - 0.01)	0.964
Hospital present	-0.02 (-0.04 - 0.01)	0.134
Park access	-0.002 (-0.01 - 0.01)	0.736
Hospital with neurological services	0.02 (0 - 0.04)	0.014

Table S4. Modeling Log standardized mortality ratio weighted by expected counts for each county.

**Variable**	**Estimate (95% CI)**	***p-value***
Age-adjusted physical inactivity	0.002 (-0.01 - 0.01)	0.598
Median household income	-0.021 (-0.03 - -0.01)	<0.001
Unemployment	0.01 (0 - 0.02)	0.211
Age-adjusted obesity	0.001 (-0.01 - 0.01)	0.9
No college degree	0.02 (0.01 - 0.03)	<0.001
Female head of household	0.013 (0 - 0.03)	0.036
Age-adjusted diabetes	0.001 (-0.01 - 0.01)	0.972
Park access	0.01 (0.01 - 0.02)	0.001
Hospital with neurological services	0.002 (-0.01 - 0.01)	0.723
Income inequality (Gini Index)	0.003 (-0.01 - 0.01)	0.531

Figure S1. Correlation matrix and heatmap of predictor variables used in the study.

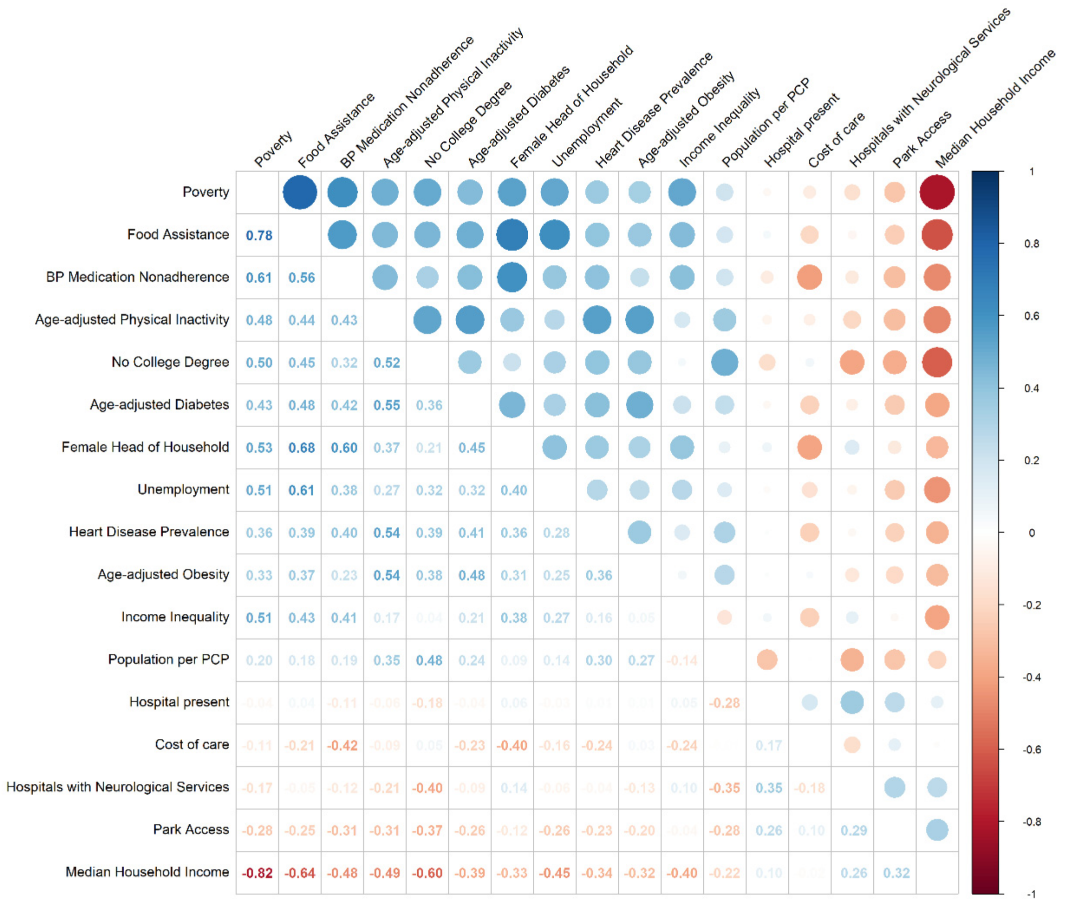

Figure S2. Map of US counties and county equivalents showing A. Prevalence of heart disease among Medicare beneficiaries, B. Blood pressure medication non-adherence, C. Cost of care among Medicare beneficiaries with heart disease, D. Age-adjusted obesity, E. Presence of hospital with neurological services, F. Presence of hospital.

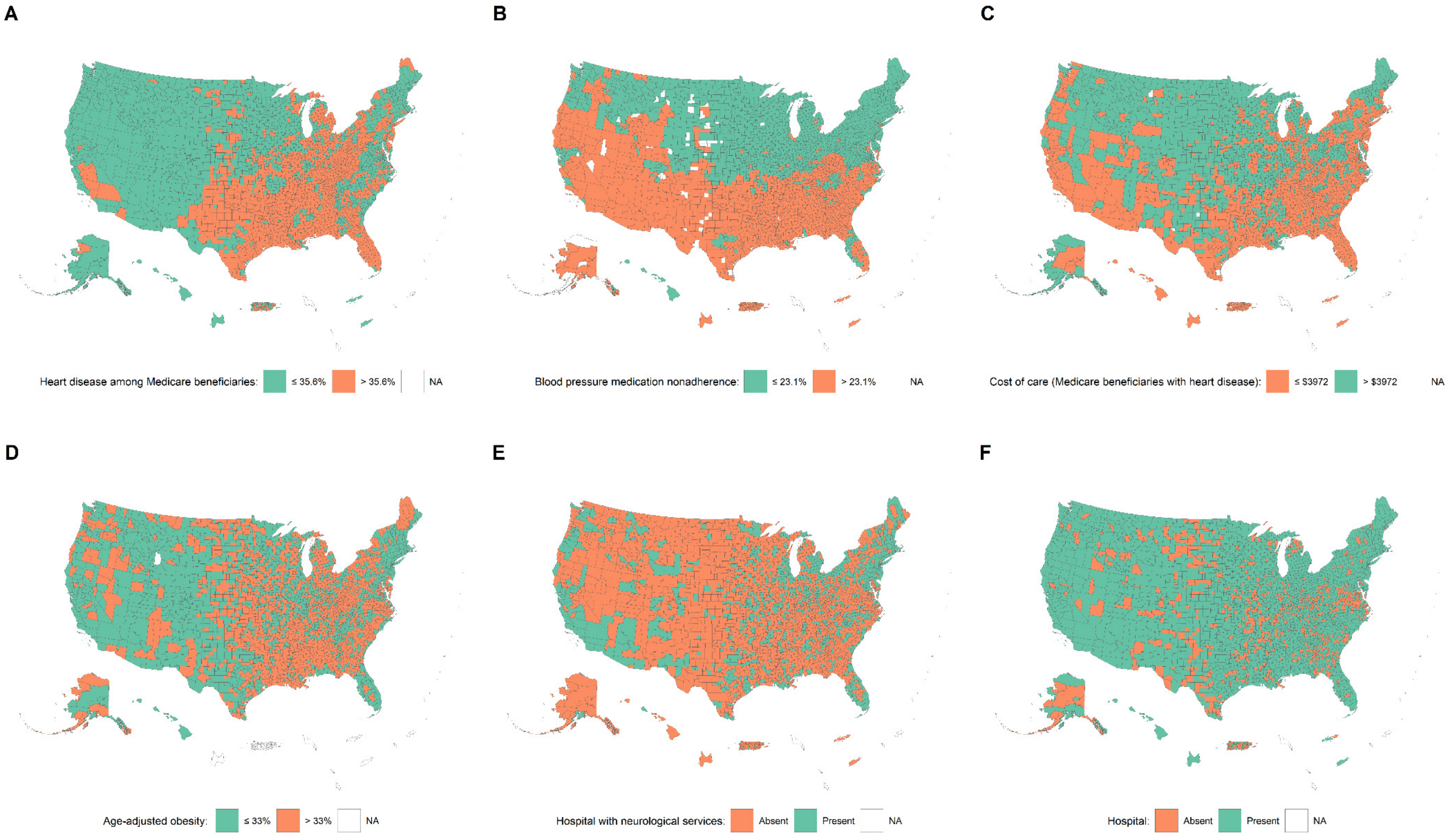

Supplement: Supplementary file 1 [file jcm-11-04101-s001.zip › jcm-1642615-supplementary.pdf]
